# Evaluation of the Potential Anti-Inflammatory Effect of a New Coumarin–Quinoline Hybrid in LPS-Induced Neuroinflammation

**DOI:** 10.3390/ph19050673

**Published:** 2026-04-25

**Authors:** Omnia Hamdy Mohamed Shehata, Eman Abdelaziz, Hadeer Ali, Elshaymaa I. Elmongy, Reem Binsuwaidan, Wafaa M. Ibrahim, Sabreen El-Gamasy, Ibrahim El Tantawy El Sayed

**Affiliations:** 1 Department of Chemistry, Faculty of Science, Menoufia University, Shebin El-Kom 32512, Egypt; 99omniahamdy@gmail.com (O.H.M.S.); emanabdelaziz24@yahoo.com (E.A.); yohader1@gmail.com (H.A.); sabreen_mohamed2008@yahoo.com (S.E.-G.); 2 Pharmaceutical Chemistry Department, Faculty of Pharmacy, Capital University (Formerly Helwan University), Ein Helwan, Cairo 11795, Egypt; shaymaa.taha@pharm.helwan.edu.eg; 3 Department of Pharmaceutical Sciences, College of Pharmacy, Princess Nourah Bint Abdulrahman University, P.O. Box 84428, Riyadh 11671, Saudi Arabia; rabinsuwaidan@pnu.edu.sa; 4 Department of Medical Biochemistry, Faculty of Medicine, Tanta University, Tanta 31511, Egypt

**Keywords:** neuroinflammation, coumarin–quinoline hybrid, LPS, NLRP3, and NF-κB

## Abstract

**Background/Objectives**: Neuroinflammation is characterized by the sustained activation of neuroglial cells, resulting in the production of cytokines and chemokines. It is associated with neurodegenerative processes. This study aims to assess the potential mitigating effect of a novel coumarin–quinoline hybrid by evaluating oxidative stress, apoptosis, and pyroptosis in an experimentally induced model of neuroinflammation. **Methods**: The study was conducted on 60 mice, allocated into six groups of ten: Group I served as the control; Group II received the new coumarin–quinoline hybrid; Group III received lipopolysaccharide (LPS); Group IV received LPS followed by the coumarin–quinoline hybrid; Group V received LPS followed by dexamethasone (DEX); and Group VI received LPS followed by the coumarin–quinoline hybrid and DEX. The model was validated by behavioral assessments, while oxidative stress was quantified via nitric oxide (NO), malondialdehyde (MDA) levels, superoxide dismutase (SOD) activity, apoptosis by caspase-3, and pyroptosis by NLRP3. **Results**: An anti-inflammatory effect of a new coumarin–quinoline hybrid, evidenced by decreased NLRP3 and NF-κB expression, reduced NO and MDA production, elevated SOD activity, and brought about suppression of caspase-3. Additionally, the newly formulated coumarin–quinoline hybrid demonstrated favorable ADMET characteristics, with in silico molecular studies indicating a stable energetic profile and dynamic equilibrium. **Conclusions**: Findings suggest that the new coumarin–quinoline hybrid holds significant potential as an adjuvant therapeutic option for neuroinflammation.

## 1. Introduction

The central nervous system (CNS) experiences an inflammatory reaction known as neuroinflammation, which is caused by the long-term activation of neuroglial cells and the ensuing release of cytokines, chemokines, and inflammatory enzymes. Numerous neurodegenerative diseases, including Alzheimer’s disease, Parkinson’s disease, multiple sclerosis, Huntington’s disease, amyotrophic lateral sclerosis, and brain cancer, have been linked to this process [1].

The outermost membrane of Gram-negative bacteria is the source of the pathogen lipopolysaccharide (LPS). It is well known for being a powerful and stable chemical that can cause a major response to inflammation in the body. LPS induces a neuroinflammatory state by activating the immune system, which results in reactive oxygen species, lipid peroxidation, oxidative brain damage, and issues with behavior and memory [2]. Toll-like receptor-4, which is widely distributed on CNS microglia, is bound by LPS to produce its effects. By activating the nuclear factor kappa B (NF-κB) pathway, this binding initiates the neuroinflammatory process [3]. A typical experimental model has been used to study the effects of inflammation of neurons on cognitive function, behavior, and neural chemistry. It is the systemic delivery of LPS to animals, even at low dosages or via a single injection. In addition to a variety of behavioral problems, including a lack of appetite, diminished physical activity, losing weight, changed exploratory behavior, increased anxiety, and disrupted sleep schedules, animals receiving LPS frequently show cognitive deficiencies [4].

Cell death is crucial for regulating inflammation and maintaining tissue homeostasis, and it can also result from inflammation. Maintaining tissue homeostasis requires both recognizing and removing dying cells [5].

The NOD-like receptor family pyrin domain-containing 3 (NLRP3) inflammasome is a vital link between innate immunity and neuroinflammation, functioning in two ways: it protects against acute inflammation while also contributing to chronic neurodegeneration. NLRP3 was the first inflammasome identified in the brain [6]. It is primarily found in microglia, but is also present in other brain cells, including oligodendrocytes, brain endothelial cells, and astrocytes. This inflammasome forms an intracellular multiprotein complex that triggers neuroinflammation in response to stress or injury [7].

A family of transcription factors called NF-κB is crucial for controlling the immune system and inflammatory responses. They regulate the production of adsorption molecules, proteins during the acute phase, chemical messengers, mediators, and inducible enzymes. This was initially thought to mediate apoptosis in immune cells. An important part of oxidative stress-related neuroinflammation is the NF-κB pathway [8].

Anti-inflammatory medications, such as glucocorticoids and non-steroidal anti-inflammatory drugs (NSAIDs), are usually used as the first line of treatment when neuroinflammation is present. Dexamethasone, a commonly used glucocorticoid, reduces blood–brain barrier permeability, acts on glucocorticoid receptors, and regulates neuroinflammation in vitro [9]. However, despite their broad anti-inflammatory effects, these drugs may not be ideal for treating neuroinflammation, as continuously high levels of plasma glucocorticoids can accelerate cognitive decline [10].

An intriguing option for creating anti-inflammatory medications is the coumarin molecule, which is present in several plants. Numerous phytoconstituents originating from the coumarin molecule, including umbelliferone, scopoletin, columbiatnetin, visniadin, marmin, and many more, have shown strong anti-inflammatory and antioxidant properties [11]. Through various mechanisms, a variety of coumarin derivatives have also been created, synthesized, and assessed for their mild to extremely strong anti-inflammatory effects. These substances have also been shown to have strong antioxidant and radical scavenging capabilities, which could strengthen their anti-inflammatory qualities [12]. Both naturally occurring coumarin derivatives and those synthesized in laboratories are known to have excellent anti-inflammatory effects. The present work demonstrates molecular insights for a new analog of coumarin derivatives having anti-inflammatory potential.

## 2. Results

### 2.1. Synthesis of Coumarin-3-Carbonyl Chloride

**Scheme 1:**
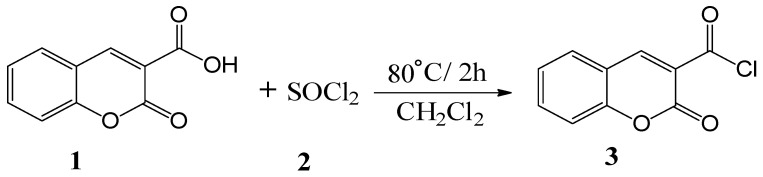
Synthesis of coumarin-3-carbonyl chloride.

As indicated in Scheme 1, the critical intermediate acid chloride **3** was produced as pale-yellow crystals in good yield by chlorinating coumarin-3-carboxylic acid **1** with thionyl chloride, SOCl_2_, in accordance with the literature approach [13].

**Scheme 2:**
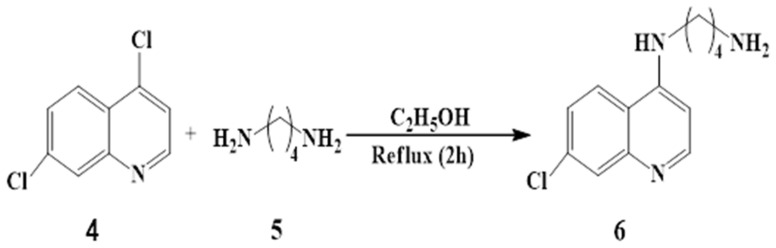
Synthesis of 4-bis-aminquinoline **6**.

As shown in Scheme 2, the nucleophilic aromatic substitution (SNAr) reaction between 4,7-dichloroquinoline **4** and 1,4-diaminobutane **5** produces 4-bis-aminoquinolines **6** in good yields using the current procedures [14,15].

**Scheme 3:**
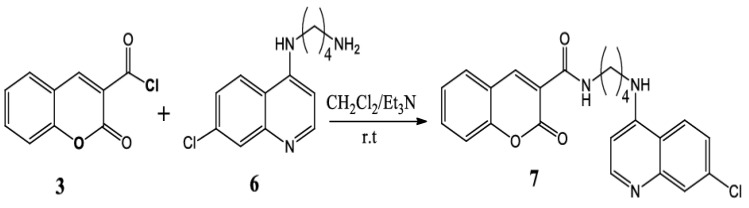
Synthesis of coumarin-based quinoline hybrid 7.

In the end, coumarin-based quinoline hybrids **7** were created by reacting coumarin-3-carbonyl chloride **3** with diamines 6 in an equimolar ratio (1:1) with triethylamine acting as a base. This produced the corresponding hybrids **7** in an excellent yield (85 percent), as shown in Scheme 3.

### 2.2. Impact of Dexamethasone and/or the New Coumarin–Quinoline Hybrid on Behavioral Tests

#### 2.2.1. Open Field Test (OFT)

**Figure 1:**
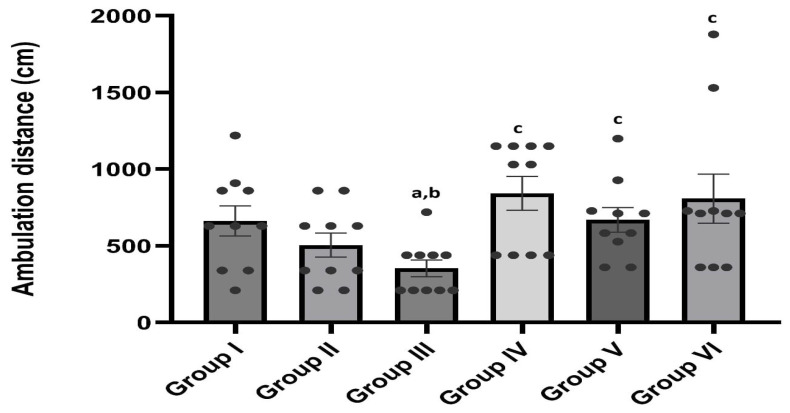
Comparison of ambulation distance (cm) among the studied groups, where ^a^ denotes a significant difference from the control group, ^b^ denotes a significant difference from Group II, and ^c^ denotes a significant difference from Group III. Group I: Control group; Group II: New coumarin–quinoline hybrid group; Group III: Lipopolysaccharide group; Group IV: Lipopolysaccharide + new coumarin–quinoline hybrid group; Group V: Lipopolysaccharide + dexamethasone group; Group VI: Lipopolysaccharide + new coumarin–quinoline hybrid + dexamethasone group.

At the beginning of the experiment, the OFT showed no significant difference in the ambulation distance values among the studied groups. However, on day 16, the ambulation distance values of the groups under study varied statistically significantly (F = 17.599, *p* = 0.0035) (Figure 1).

#### 2.2.2. Novel Object Recognition (NORT)

**Figure 2:**
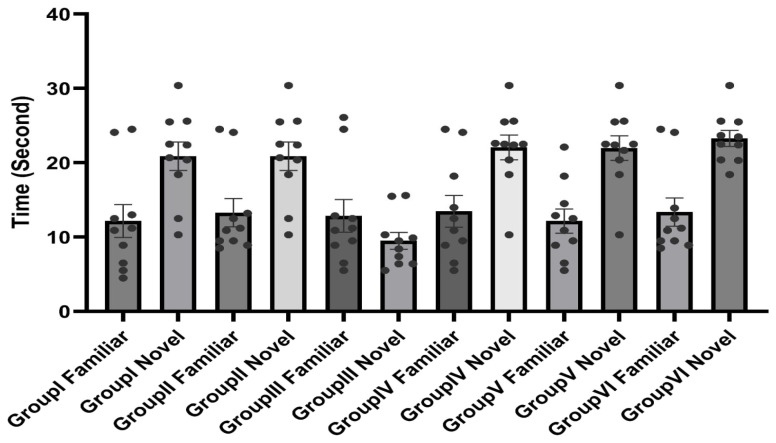
Comparison of NORT performance, expressed as exploration time (seconds) among the studied groups. **Group I**: Control group; **Group II**: New coumarin–quinoline hybrid group; **Group III**: Lipopolysaccharide group; **Group IV**: Lipopolysaccharide + new coumarin–quinoline hybrid group; **Group V**: Lipopolysaccharide + dexamethasone group; **Group VI**: Lipopolysaccharide + new coumarin–quinoline hybrid + dexamethasone group.

At the beginning of the experiment, NORT showed no significant difference among the studied groups. On days 17 and 18, NORT exhibited a statistically significant difference (F = 19) in the total exploration time values between the two objects among the groups under study (959, *p* < 0.001) (Figure 2).

### 2.3. Impact of Dexamethasone and/or the New Coumarin–Quinoline Hybrid on Oxidative Stress Parameters

MDA levels varied significantly among the groups under study, with the LPS group (Group III) exhibiting higher MDA levels than the control group. MDA levels significantly decreased in comparison to the LPS group when the new coumarin–quinoline hybrid and/or dexamethasone were administered; Group VI’s combined treatment produced the largest reduction. The LPS group’s SOD activity was significantly lower than that of the control group. In comparison to the LPS group, treatment with the new coumarin–quinoline hybrid and/or dexamethasone markedly increased SOD activity; the combined treatment group demonstrated even more improvement. Significant differences were also noted for nitric oxide concentration, with the LPS group exhibiting lower levels than the control group (Table 1).

### 2.4. Effect of the New Coumarin–Quinoline Hybrid and/or Dexamethasone on Caspase-3

**Figure 3:**
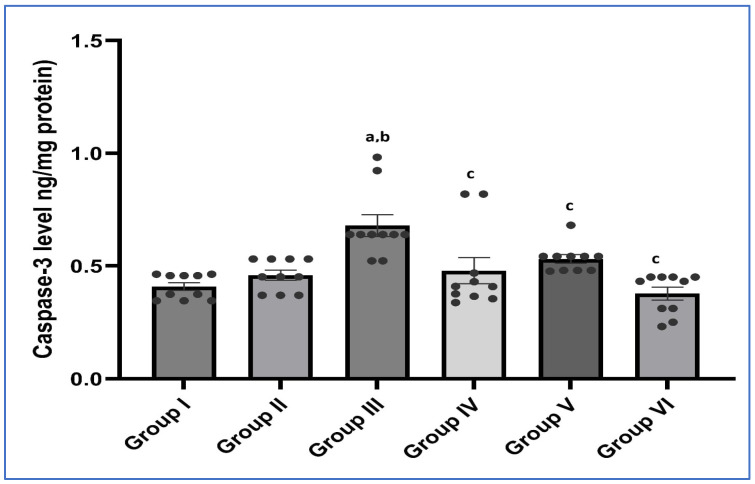
Comparison of caspase-3 levels (ng/mg protein) in brain tissue among the studied groups, where ^a^ denotes a significant difference from the control group, ^b^ denotes a significant difference from Group II, and ^c^ denotes a significant difference from Group III. Group I: Control group; Group II: New coumarin–quinoline hybrid group; Group III: Lipopolysaccharide group; Group IV: Lipopolysaccharide + new coumarin–quinoline hybrid group; Group V: Lipopolysaccharide + dexamethasone group; Group VI: Lipopolysaccharide + new coumarin–quinoline hybrid + dexamethasone group.

The LPS group (Group III) showed a statistically significant increase in the caspase-3 level when compared to the control group (Group I). Nevertheless, new coumarin–quinoline hybrid and/or dexamethasone treatments ameliorated apoptosis and revealed a statistically significant decrease in the caspase-3 level when compared to the LPS group. Remarkably, the combined treatment group (Group VI) demonstrated an obvious recovery with a more pronounced reversal in the caspase-3 level, which was even more significant than that of the control group (Group I) (Figure 3).

### 2.5. Effect of New Coumarin–Quinoline Hybrid and/or Dexamethasone on NF-kB and NLRP3 Relative Gene Expression

**Figure 4:**
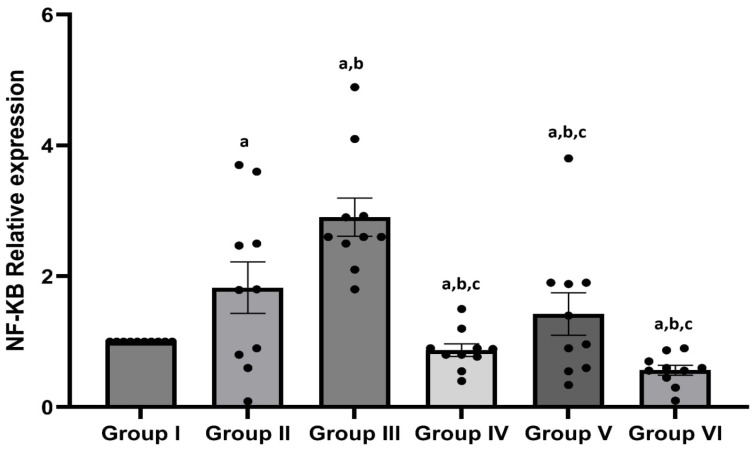
Comparison of NF-kB relative gene expression in brain tissue among the studied groups, where ^a^ denotes a significant difference from the control group, ^b^ denotes a significant difference from Group II, and ^c^ denotes a significant difference from Group III. Group I: Control group; Group II: New coumarin–quinoline hybrid group; Group III: Lipopolysaccharide group; Group IV: Lipopolysaccharide + new coumarin–quinoline hybrid group; Group V: Lipopolysaccharide + dexamethasone group; Group VI: Lipopolysaccharide + new coumarin–quinoline hybrid + dexamethasone group.

Findings have demonstrated that nuclear factor-kB (NF-kB) relative gene expression among the groups under investigation varied statistically significantly in certain aspects of neuroinflammation. NF-kB relative gene expression was statistically significantly higher in the LPS group (Group III) than in the control group (Group I). However, compared to the LPS group, the new coumarin–quinoline hybrid and/or dexamethasone treatments showed a statistically significant reduction in NF-kB relative gene expression. Surprisingly, compared to the control group (Group I), the combined treatment group (Group VI) showed a clear recovery with a more noticeable reversal in NF-kB relative gene expression (Figure 4).

**Figure 5:**
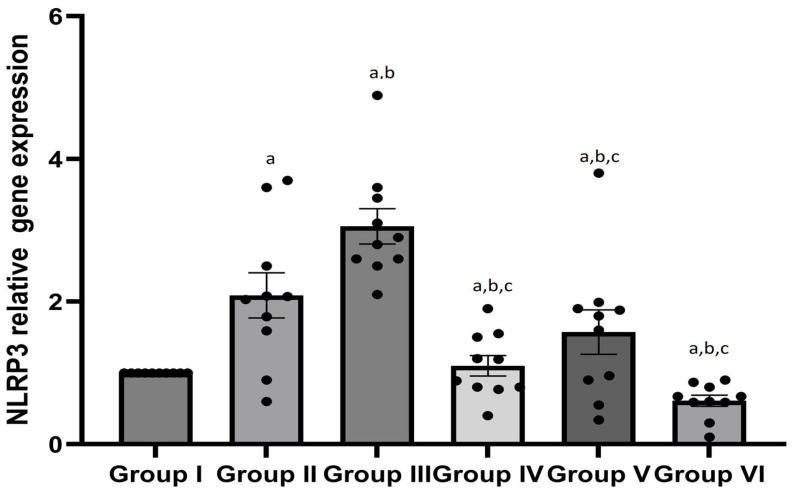
Comparison of NLRP3 relative gene expression in brain tissue among the studied groups, where ^a^ denotes a significant difference from the control group, ^b^ denotes a significant difference from Group II, and ^c^ denotes a significant difference from Group III. Group I: Control group; Group II: New coumarin–quinoline hybrid group; Group III: Lipopolysaccharide group; Group IV: Lipopolysaccharide + new coumarin–quinoline hybrid group; Group V: Lipopolysaccharide + dexamethasone group; Group VI: Lipopolysaccharide + new coumarin–quinoline hybrid + dexamethasone group.

There was a statistically significant difference in the relative gene expression of NLRP3 among the study groups. NF-kB relative gene expression was statistically significantly higher in the LPS group (Group III) than in the control group (Group I). However, compared to the LPS group, the new coumarin–quinoline hybrid and/or dexamethasone treatments showed a statistically significant reduction in the relative gene expression level of NLRP3. Surprisingly, compared to the control group (Group I), the combined treatment group (Group VI) showed a clear recovery with a more noticeable reversal in NLRP3 relative gene expression (Figure 5).

### 2.6. Histological Finding

**Figure 6:**
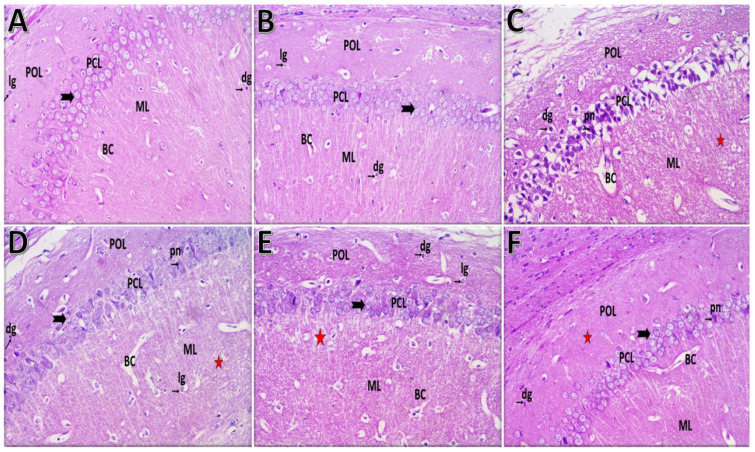
A photomicrograph of the hippocampal cornu Ammonis 1 region of the groups under study. (**A**) The control group revealed three distinct layers: the polymorphic layer (POL), pyramidal cell layer (PCL), and molecular layer (ML). The cell bodies of the pyramidal neurons (bifid black arrow) in PCL are closely packed, regularly arranged in three to four rows, and appear small with vesicular nuclei, prominent nucleoli, and little cytoplasm. The nuclei of glial cells with normal blood capillaries (BC) are stained deeply (dg) and lightly (lg) in the POL and ML. (**B**) The histological structure of the new coumarin–quinoline hybrid Group II was normal and comparable to that of the control group. (**C**) LPS Group III displayed dilated blood capillaries (BCs), perinuclear halo surrounding deeply stained glial cell nuclei (dg), vacuolated neuropils (red star), fewer pyramidal cells (PCLs), and the majority of pyramidal neurons with pyknotic nuclei (pn). (**D**) Group IV treated with LPS + new coumarin–quinoline hybrid showed improvement of histological architecture like the control group, except for dilated blood capillary (BC), a few pyknotic nuclei of pyramidal cells (pn), and mild vacuolated neuropil (red star). (**E**) LPS + Dexa Group V revealed histological structure like the control group except for mild vacuolated neuropil, which is seen (red star). (**F**) LPS + Dexa + new coumarin–quinoline hybrid Group VI revealed marked improvement of histological architecture, like the control group, except for mild vacuolated neuropil, which is seen (red star). (H&E Mic. Mag. × 400).

Group I (control) and Group II (new coumarin–quinoline hybrid) showed normal histological structures in the hippocampus, with well-defined layers, closely packed pyramidal neurons, and normal glial cell nuclei. Group III (LPS) exhibited significant histological disturbance, including vacuolated neuropil and pyknotic nuclei in pyramidal neurons, along with dilated blood capillaries. Group IV (LPS + new coumarin–quinoline hybrid) demonstrated moderate recovery, while Group V (LPS + Dexa) showed some improvements. Group VI (LPS + new coumarin–quinoline hybrid + Dexa) reflected marked improvements, resembling the control group’s structure (Figure 6).

### 2.7. In Silico Pharmacokinetic Assessment

In the early stages of drug development, in silico ADME (absorption, distribution, metabolism, and excretion) studies are crucial [16,17,18,19]. Key absorption-related properties—such as intestinal absorption, skin sensitization, and oral bioavailability—were evaluated (Table 2). The hybrid compound 7 showed intestinal absorption exceeding the commonly cited 30% threshold, with an observed absorbance value of 91%.

All tested compounds exhibited skin permeability, with a score of −2.7 cm/h. Additionally, compound **7** demonstrated moderate permeability in the human colon adenocarcinoma (Caco-2) model, with a score of 0.86 relative to the reference standard. In this reference, permeability is considered high when the Caco-2 value is greater than 0.9 [16,19].

For distribution, the in silico assessment focused on volume of distribution (VDss), blood–brain barrier (BBB) permeability, and central nervous system (CNS) permeability. The VDss results suggested distribution volumes of 0.1 log L/kg, while the log BB score was low at −0.53, as log BB will reflect low BBB if <−1 [16].The log permeability-surface (log PS) value for CNS permeability was −2.07, which is a good permeability indication when compared to the reference, stating that a low CNS permeability is when log PS < −3 [16]. Hepatic and renal clearance were also estimated to gauge overall drug clearance, based on total clearance and elimination rate. Excretion was reported as log (mL/min/kg). The expected ADME findings are summarized in the results provided in Table 2.

## 3. Discussion

Neuroinflammation arises from immune responses in the CNS, linked to conditions like depression and Alzheimer’s. Gram-negative bacteria’s LPS triggers pro-inflammatory responses. Dexamethasone is effective but risky. Coumarins, plant-derived compounds, exhibit significant anti-inflammatory, anticoagulant, antioxidant, antimicrobial, and anticancer activities, highlighting their therapeutic potential [20]. So, this study aims to assess the possible anti-inflammatory effect of a new coumarin–quinoline hybrid in experimentally induced neuroinflammation.

Results have shown that the functional effects of LPS on rodent locomotion and anxiety-like behavior were evaluated in a neurobehavioral study using the Novel Object Recognition Test (NORT) and Open Field Test (OFT) [21]. Altered neurological processes and possibly aberrant brain function are indicated by changes in locomotion function [22], mice’s recognition memory functions [23]. In comparison to the control group, the LPS group exhibited a statistically significant reduction in OFT and NORT, according to the current study. Furthermore, compared to the LPS group, the dexamethasone-treated group also displayed a statistically significant decline. Our LPS administration results are consistent with earlier research that demonstrated that systemic LPS treatment causes anxiety-like behavior in rodents and decreases spontaneous locomotor activity [22,23]. Our LPS administration results are consistent with earlier research [24,25,26]. In addition, our results of dexamethasone treatment were in coherence with a previous study [27]. On the other hand, mice treated with the combination of the new coumarin–quinoline hybrid and dexamethasone showed an increase in ambulation distance, suggesting that the new coumarin–quinoline hybrid had greatly reduced anxiety-like behavior and the adverse effects of dexamethasone [28].

Oxidative stress caused by the production of reactive oxygen species (ROS) has a significant impact on inflammatory processes. The body can frequently use the enzymes of the antioxidant defense system to get rid of excessive free radicals to prevent oxidative damage [29]. Antioxidant enzyme activity and MDA levels can be used to assess oxidative damage. The antioxidant enzyme system’s initial line of defense against ROSs generated during oxidative stress is thought to be SOD [30].

The current study showed a statistically significant rise in MDA levels following LPS treatment. This is consistent with the findings of Essadek et al. (2022) who found that intraperitoneal injection of LPS significantly increased cerebral MDA production in mice after 4 h [31]. Indeed, the binding of LPS to TLR4 triggers a downstream signaling pathway that concludes with IκB phosphorylation and NF-κB nuclear translocation. In turn, activated NF-κB up-regulates the expression of pro-inflammatory genes and ROS-producing enzymes. The increased ROS oxidizes polyunsaturated fatty acids to produce MDA [32]. The new coumarin–quinoline hybrid and dexamethasone combination significantly reduced MDA levels compared to the lipopolysaccharide group, indicating enhanced effectiveness in mitigating oxidative stress and free radical reactions during inflammation treatment, potentially due to suppressing oxidation pathways, reducing lipid peroxidation, and modulating antioxidant and prooxidant enzyme activities [33].

Brain superoxide dismutase (SOD) regulates oxidative stress. The LPS-treated group exhibited significantly reduced SOD activity compared to the control, likely due to oxidative damage from excessive free radicals affecting enzyme protein quality [34]. Previous reports indicated that LPS decreased the activities of antioxidant enzymes such as SOD [35]. Additionally, LPS is one of the compounds capable of inducing oxidative stress in different cells of different organs, leading to the production of toxins [36]. On the other hand, the new coumarin–quinoline hybrid treatment and dexamethasone treatment increased and improved the activity of SOD when compared to the LPS-treated group. The results herein are consistent with those obtained previously [37,38]. Better improvement was in the group that received the combined new coumarin–quinoline hybrid and dexamethasone treatment. So, this combination has a better antioxidant effect than a single administration of the new coumarin–quinoline hybrid or dexamethasone.

Nitric oxide (NO) is derived from L-arginine via NO synthase (NOS) [39]. The LPS-treated group showed decreased NO levels significantly compared to controls, suggesting potential protective anti-inflammatory activities through antioxidant properties. Othman et al. (2020) determined that lipid peroxidation and nitric oxide increased while glutathione decreased in rats treated with 3-benzoyl-7-hydroxy coumarin [40]. The use of coumarin derivatives enhances the anti-inflammatory effect of DEX and may potentiate the activity of each other.

Caspase-3 plays crucial roles in tissue differentiation, regeneration, and neural development, with dysregulated apoptosis linked to various pathological conditions [41]. In the current study, the LPS-treated group showed a statistically significant increase in the caspase-3 level when compared to the control group. This was in harmony with the previous study [42], which revealed that the treatment of LPS resulted in a significant increase in cleaved caspase-3 expression compared to the control level. This might be because LPS activates the TLR4 signaling pathway, which triggers the inflammatory response and produces NO and ROS. These proinflammatory mediators function as secondary messengers that trigger signal transduction and control the genes that trigger inflammatory cytokines, which influence inflammation and apoptosis [43]. Another study showed that 3-benzoyl-7-hydroxy coumarin significantly reduced caspase-3 and TNF-α levels and had protective effects in the tissue by suppressing apoptosis [44].

Proinflammatory gene transcription is induced by NF-κB activation via both canonical and noncanonical mechanisms [45].

The present results have indicated a significant difference in NF-kB relative gene expression among groups. The LPS group (III) exhibited increased NF-kB expression compared to the control group (I). Treatment with the new coumarin–quinoline hybrid and/or dexamethasone led to a significant decrease in NF-kB expression relative to the LPS group (L). The combined treatment group (VI) showed notable recovery, with NF-kB expression levels significantly lower than those of the control group (I). In its inactive state, the NF-κB complex binds to IκB protein, preventing nuclear translocation. IκB activation promotes NF-kB release, leading to increased production of inflammatory cytokines like IL-6, IL-1β, and TNF-α [46]. In line with our results, Zhao et al. (2023) elucidated that LPS could cause inflammation and increase NF-κB expression by inhibiting the expression of Nrf2 and HO-1 [47].

Dexamethasone was hypothesized to mitigate inflammatory reactions in the CNS [48,49], indicating that the TLR-4/NF-kB pathway has a critical role in brain injury. DEX treatment led to significantly reduced levels of TLR-4 and NF-kB, highlighting its neuroprotective effects.

NLRP3, ASC, and caspase-1 make up the multiprotein complex known as the NLRP3 inflammasome, which reacts to danger signals and infections. When it is activated, pyroptosis mediated by caspase-1 occurs, and IL-1β and IL-18 are released, essential for immune defense. However, inappropriate activation is linked to diseases like diabetes, cancer, and Alzheimer’s [50].

According to the results of the present work, the LPS-treated group’s NLRP3 expression was noticeably higher than that of the control group. Given that LPS has been shown to significantly increase inflammation via the TLR4/NF-κB pathway, which encourages the transcription of inflammatory genes like NLRP3, this is not shocking.

Treatment with either the new coumarin–quinoline hybrid or dexamethasone showed significantly decreased NLRP3 expression compared to that in the LPS group. This can be brought about by the anti-inflammatory properties of both agents: dexamethasone, which inhibits NF-κB activity and suppresses the transcription of pro-inflammatory genes, and the new coumarin–quinoline hybrid, which has antioxidant properties and decreases the production of ROS, thereby weakening the activation of the NLRP3 inflammasome [51,52,53]. These findings indicate that both treatments might suppress the inflammatory response and prevent cell injury associated with excessive NLRP3 activation, supporting their use as protective or therapeutic agents in inflammatory conditions.

The neuroanatomical changes in the hippocampus of different experimental groups are reflective of six-group comparative studies that have presented clear and convincing evidence of the two processes: injury and neuroprotection in a lipopolysaccharide (LPS)-induced inflammation model. The hippocampal CA1 region of the brain in the control group (Group I) and the coumarin–quinoline hybrid alone-treated group (Group II) showed intact architecture, tightly packed pyramidal layers, vesicular nuclei, and brightly stained nucleoli in the pyramidal neurons, as well as the normal form of glial nuclei and capillaries. This demonstrates that the molecule is not disrupting normal neuroanatomy and, therefore, it could be a safe baseline [54].

On the contrary, the group injected with LPS alone (Group III) displayed significant neuropil vacuolation, a decrease in pyramidal cell density, and the presence of pyknotic nuclei—the features that are most typical for the neuroinflammatory injury caused by the activation of microglia, the release of cytokines (e.g., TNF-α, IL-1β), and the production of reactive oxygen species (ROS) leading to the neuronal apoptosis or necrosis [55].

In the treatment groups, the extent of the hippocampal tissue restoration ranged from moderate to significant. Cellular architecture of the brain was partially restored in Group IV (LPS + hybrid), with some residual neuropil vacuolation and occasional pyknotic cells, thus demonstrating that the hybrid compound has neuroprotective potential under inflammatory conditions. This finding is in line with the literature, indicating the antioxidant, anti-inflammatory, and anti-apoptotic effects of coumarin and quinoline derivatives, along with their BDNF/TrkB-enhancing and caspase pathway down-regulating effects [54,55,56]. The recovery in Group V (LPS + dexamethasone) was even more pronounced, and this observation could be explained by the corticosteroid’s ability to alleviate neuroinflammation through the inhibition of microglial activation and the release of pro-inflammatory cytokines [57]. Importantly, Group VI (LPS + hybrid + dexamethasone) showed almost complete restoration of the hippocampal structure with all layers being recognizable, nucleic morphology of pyramidal neurons being normal, and vascular elements being re-established. Such synergistic effects suggest that the combination of the anti-inflammatory capacity of dexamethasone with the neuroprotective pathways of the hybrid compound is the reason for the alleviation of hippocampal damage.

These data highlight three major points: firstly, that the hippocampal lesions, which are inflammatory in nature and caused by LPS, are extensive and show a very close resemblance to neuroinflammation; secondly, that the coumarin–quinoline hybrid is potent enough to mitigate the structural damage; and thirdly, that adding this hybrid to dexamethasone leads to the greatest protective effect. From a clinical point of view, the effect of targeting oxidative stress and inflammation early on is likely to result in the preservation of the hippocampal neurons that are still viable in the event of a systemic endotoxin challenge.

## 4. Materials and Methods

### 4.1. Materials Used to Prepare New Coumarin–Quinoline Hybrid

4,7-Dichloroquinoline 97% (Sigma Aldrich, St. Louis, MO, USA), 1,4-diaminobutane 99% (LOBA Chemie, Mumbai, India), ethanol 98% (Sigma Aldrich, St. Louis, MO, USA) dichloromethane 98% (LOBA Chemie, Mumbai, India), trimethylamine 99% (Sigma Aldrich, St. Louis, MO, USA), coumarin-3-carboxylic acid 99% (Sigma Aldrich, St. Louis, MO, USA), thionyl chloride 97% (LOBA Chemie, Mumbai, India), petroleum ether (LOBA Chemie, Mumbai, India).

### 4.2. Instrumental Characterization

^1^H-NMR and ^13^C-NMR spectra were recorded on Bruker spectrometer (Karlsruhe, Germany) in DMSO-d6 (500 MHz for 1H-NMR and 125 MHz for ^13^C-NMR). FTIR analyses were performed with Alpha, Bruker Germany (Mannheim, Germany). Mass spectrometry was analyzed using Agilent G1946D LC/MS, Santa Clara, CA, USA. HPLC was analyzed using an Agilent 1100 liquid chromatography integrated system equipped with G1313A automated injector, G1311A quaternary pump, and G1315B diode-array detector (DAD) operated at a wavelength of 260 and 330 nm. The chromatographic separation of the compounds was achieved with a reversed-phase column, ZORBAX XDB-C18 (5.0 × 250, 5 µm), from Agilent (Santa Clara, CA, USA) operating at constant temperature (30 °C). The 10 mg samples were dissolved in 10 mL of eluent consisting of water: methanol (50:50), filtered through a 0.45 µm nylon filter, and injected into the HPLC. The samples were eluted at a flow rate of 1 mL/min, and a linear gradient started by 10% water and 90% methanol to 90% water and 10% methanol after 20 min.

### 4.3. General Synthesis of Coumarin-Based Quinoline Hybrid 7

Coumarin-3-carbonyl chloride **3** (0.30 g, 1.27 mmol) and 1,4-diaminobutane 6 (0.33 g, 1.27 mmol) were dissolved in CH_2_Cl_2_ (2 mL). Triethylamine (0.39 g, 3.81 mmol) was added with stirring at 25 °C. The reaction was followed by thin-layer chromatography (TLC) until materials were exhausted for 12 h. The mixture was spilled out into ice/water, and extracted three times with CH_2_ Cl_2_; the organic layer was collected, dried, and then removed of the solvent residue using a rotatory evaporator. The precipitate was filtered, dried, and then recrystallized from ethanol to obtain pure **7** in good yield, 85%.

#### Synthesis of N-(4-(7-Chloroquinolin-4-Yl)Amino)Butyl)-2-oxo-2H-Chromene-3-Carboxamide

White solid, yield (0.45 g, 85%). FT-IR (KBr) cm^−1^ ʋ: 3437 (NH), 2931 (CH), 1713 (C-C=O), 1606 (C=CAr), 1552 (C=N), 1289 (C-C) as shown in Figure S1. ^1^H-NMR (DMSO-d6 500 MHz) δ ppm: 1.215 (m, 2H, CH2), 1.665 (m, 2H, CH2), 3.024 (m, 2H, CH2), 3.34 (m, 2H, CH2), 7.77–8.87 (m, 10H, CHAr) as shown in Figure S2. ^13^C-NMR (DMSO-d6 100 MHz) δ ppm: 25.49, 27.00, 42.65, 52.13, 99.12, 116.57, 117.39, 118.89,119.39, 122.54, 124.81, 125.65, 126.36, 129.22, 130.46, 133.66, 134.59, 135.93, 147.74, 154.22, 160.80, 161.66 as shown in Figure S3; HPLC purity: >99% at (Rt) = 16.566 min as shown in Figure 4. HRMS(ESI): *m*/*z* (C_23_H_20_ClN_3_O_3_) calcd, 421.88; found, 422.3 [M^+^], 424.3 [M + 2]^+^ as shown in Figure S5.

### 4.4. Synthesis of a New Coumarin–Quinoline Hybrid and Drug Preparation

Synthesis of the new coumarin–quinoline hybrid was achieved according to the literature [58]. The new coumarin–quinoline hybrid was dissolved in a mixture of dimethyl sulfoxide (DMSO) and saline (9% normal saline) at a ratio of 1:1, with a dosage of 1 mg/kg. LPS (*Escherichia coli* 055: B5, purified by phenol extraction, Lot No. 1031 Q033), purchased from Solar Bio–Life Science in Beijing, China, was used to induce recognition memory impairment in mice. A stock solution of LPS was prepared at a concentration of 10 mg/mL by dissolving 10 mg of LPS powder in 1 mL of saline. This solution was then divided into 100 µL aliquots and stored at −20 °C. All injections were freshly prepared from the stock solution to achieve a dosage of 250 µg/kg/day via intraperitoneal (i.p.) administration.

### 4.5. Animals

This study was conducted using 60 mice (BALB/C), with weights ranging from 20 to 25 g, sourced from the Theodore Billiharz Institute for Research in Giza, Egypt. A regulated setting with a temperature of 23 ± 2 °C and a humidity of 65% was utilized to accommodate ten mice in each cage. The mice were maintained at room temperature under a 12 h light/dark schedule, and they received regular food and water. All experiments were carried out following the guidelines, rules, and regulations set by the Institutional Animal Care and Use Committee (IACUC), Faculty of Science, Menoufia University (approval no. MUFS/S/Bio/2/25).

### 4.6. Experimental Design

At random, the animals were allocated into six groups of ten mice each. (I) A vehicle (0.9 percent normal saline intraperitoneally) was administered to healthy mice in the control group for 14 days; (II) the new coumarin–quinoline hybrid group received a new coumarin–quinoline hybrid (1 mg/kg/day, i.p.) for 14 days; (III) the LPS group received LPS (250 µg/kg/day, i.p.) for 7 days followed by a vehicle (0.9% normal saline. i.p.) for 7 days; (IV) LPS + new coumarin–quinoline hybrid group received LPS (250 µg/kg/day, i.p.) for 7 days followed by a new coumarin–quinoline hybrid (1 mg/kg/day, i.p.) for 7 days; (V) LPS + DEX received LPS (250 µg/kg/day, i.p.) for 7 days followed by DEX (1 mg/kg/day, i.p.) for 7 days; (VI) LPS + new coumarin–quinoline hybrid + DEX group received LPS (250 µg/kg/day, i.p.) for 7 days followed by a new coumarin–quinoline hybrid (1 mg/kg/day, i.p.) and DEX (1 mg/kg/day, i.p.) for 7 days.

All the study groups undergo behavioral tests, OFT, and NORT twice: once at the beginning of the experiment and again on days 16, 17, and 18 to evaluate recognition memory.

### 4.7. Behavioral Examinations

#### 4.7.1. The Open Field Test (OFT)

OFT assesses locomotor activity, anxiety, and exploration in mice. Mice explore a clean arena for 5 min, after which they are returned home. Corner seating leads to exclusion, while total distance moved and velocity are used to evaluate performance [59].

#### 4.7.2. The Novel Object Recognition Test (NORT)

NORT assesses mice’s recognition memory influenced by LPS, dexamethasone, and a coumarin derivative across two days. Mice participate in habituation, familiarization, and discrimination sessions [59].

### 4.8. Brain Tissue Homogenates

Mice were anesthetized by i.p. injection of ketamine (75 mg/kg) and xylazine (10 mg/kg) on day 21. Following scarification, brain tissues were meticulously dissected, evaluated, and given three ice-cold saline washes to remove debris before being blotted one at a time on filter paper free of ash. The three pieces of each specimen were weighed, and the first piece was homogenized at a ratio of 100 mg of tissue to 1 mL of buffer using a Potter-Elvehjem tissue homogenizer (20–30 up and down strokes) for biochemical examination. The other piece was homogenized in triazole for other biochemical determination. The third piece was preserved in formalin for histological examination.

### 4.9. Biochemical Parameters

Brain tissue homogenate was utilized to determine the malondialdehyde (MDA) concentration [60], superoxide dismutase (SOD) activity using a commercial kit provided by Biokit Egypt (Catalog number: SD 25 21), nitric oxide (NO) concentration using a commercial kit (Biodiagnostic) supplied by Biokit Egypt (Alexandria, Egypt, catalog number: 240222), and caspase-3 concentrations using a mouse caspase-3 ELISA kit, Sunred (Shanghai, China, catalog number: 201-02-0446).

### 4.10. Determination of Gene Expression

The GeneJET RNA Purification Kit (Thermo Scientific, Waltham, MA, USA) was used to extract total RNA from brain tissue. The RNA concentration and purity were evaluated using a Thermo Scientific™ Evolution™ 201/220 UV–visible spectrophotometer (Thermo Fisher Scientific, Waltham, MA, USA). Genomic DNA was extracted from 1 μL of isolated RNA using a 4× gDNA wiper mix. To create cDNA, 4 μL of 5× qRT Super Mix II was added to the reaction mixture, and it was then incubated at 50 °C for 15 min. The SYBR Green Master Mix real-time quantitative PCR kit was employed. To ascertain the final PCR quality evaluation product, a melting curve analysis was carried out at the conclusion of the primer reaction (Table 3). A SYBR Green Master Mix kit was used to prepare the quantitative PCR. For each sample, the C(t) values were computed three times. As an internal reference control, 18S rRNA was employed as shown in Figure S6.

### 4.11. Histological Examination

Brain tissues were preserved in 10% neutral buffered formalin for 48 h. After fixation, 2 mm-thick sagittal brain sections were created and converted into paraffin blocks. These blocks were cut into 5 µm slices using a rotary microtome. The paraffin sections were placed on glass slides and dyed with hematoxylin and eosin. Histopathological examination was performed using a light microscope, and images were captured (Figures S7 and S8).

### 4.12. Statistical Analysis

A Shapiro–Wilk test (*p* > 0.05) [61,62] has been used. A visual inspection of their histograms, normal Q–Q plots, and box plots showed that our results were approximately normally distributed for all parameters. One-way analysis of variance (ANOVA) was used to assess significant differences among treated groups. The Tukey test was used to compare all groups with each other and shows the significant effect of treatment.

### 4.13. In Silico Pharmacokinetic

In silico studies were performed using pre-ADMET software pkcsm (https://biosig.lab.uq.edu.au/pkcsm/, accessed on 23 March 2026).

## 5. Conclusions

In conclusion, the new coumarin–quinoline hybrid exhibited mitigating effects against LPS-induced neuroinflammation. The compound has significantly reduced the severity of neuroinflammation by controlling oxidative stress, apoptosis, and inflammatory pathways, particularly via regulating NF-KB expression. These findings were supported by the observed improvement in histopathological results as well as behavioral impairments. Additionally, the results suggested that the newly synthesized coumarin–quinoline hybrid showed significant promise as an effective choice for the management of neuroinflammation. Accordingly, the effect of targeting oxidative stress and inflammation early on is likely to result in the preservation of the neurons that are still viable in the event of a systemic endotoxin challenge. Moreover, ADME analysis confirmed its promising pharmacokinetic properties.

## Figures and Tables

**Table 1:** Statistical comparison of oxidative stress parameters in brain tissue among the studied groups.

		Group I (*n* = 10)	Group II (*n* = 10)	Group III (*n* = 10)	Group IV (*n* = 10)	Group V (*n* = 10)	Group VI (*n* = 10)
MDA level (nmol/mg protein)	Range	0.49–0.97	0.49–0.91	1.49–8.92	0.24–3.54	1.07–2.67	0.19–0.84
	Mean ± SE	0.67 ± 0.06	0.68 ± 0.05	4.5 ± 0.4 ^a,b^	1.7 ± 0.4 ^c^	1.6 ± 0.2 ^c^	0.56 ± 0.09 ^c^
SOD activity (U/mg protein)	Range	42.8–44.4	28.7–48.7	14.3–35.2	43.6–78.74	27–44.7	29.4–81.3
	Mean ± SE	43.6 ± 0.18	40.7 ± 1.6	28.3 ± 1.9 ^a,b^	52.8 ± 3 ^c^	35.7 ± 1.8 ^c^	48.7 ± 5.5 ^c^
Nitric oxide (μmol/mg protein)	Range	1.8–2.17	1.6–1.8	1.19–9.2	1.14–2.62	1.3–6.9	0.66–1.663
	Mean ± SE	2.04 ± 0.04	1.75 ± 0.04	3.2 ± 0.8 ^a,b^	1.3 ±0.14 ^c^	2.88 ± 0.6	1.2 ± 0.2 ^c^

*n*: number of mice; SE: standard error; *p*-value < 0.05 is significant, where ^a^ denotes a significant difference from the control group, ^b^ denotes a significant difference from Group II, and ^c^ denotes a significant difference from Group III. Group I: Control group; Group II: New coumarin–quinoline hybrid group; Group III: Lipopolysaccharide group; Group IV: Lipopolysaccharide + new coumarin–quinoline hybrid group; Group V: Lipopolysaccharide + dexamethasone group; Group VI: Lipopolysaccharide + new coumarin–quinoline hybrid + dexamethasone group.

**Table 2:** In silico screening of ADMET properties for the hybrid compound 7.

Property	Model Name (Unit)	Predicted Value
		Compound 7
Absorption	Intestinal absorption (human) (% absorbed)	91.037%
	Caco2 permeability (log Papp in 10^−6^ cm/s)	0.868
	Water solubility (log mol/L)	−5.019
	Skin permeability (log Kp)	−2.738
	P-glycoprotein substrate	Yes
	P-glycoprotein I inhibitor	Yes
	P-glycoprotein II inhibitor	Yes
Distribution	VDss (human) (log L/kg)	0.1
	Fraction unbound (human) (Fu)	0.057
	BBB permeability (log BB)	−0.533
	CNS permeability (log PS)	−2.076
Metabolism	CYP2D6 substrate	Yes
	CYP3A4 substrate	Yes
	CYP1A2 inhibitor	Yes
	CYP2C19 inhibitor	Yes
	CYP2C9 inhibitor	Yes
	CYP2D6 inhibitor	No
	CYP3A4 inhibitor	Yes
Excretion	Total clearance (log ml/min/kg)	0.045
	Renal OCT2 substrate	No
Toxicity	AMES toxicity	No
	Max. tolerated dose (human) (log mg/kg/day)	0.564
	hERG I inhibitor	No
	hERG II inhibitor	Yes
	Oral rat acute toxicity (LD50) (mol/kg)	2.238
	Oral rat chronic toxicity (LOAEL) (log mg/kg_bw/day)	1.428
	Hepatotoxicity	Yes
	Skin sensitization	No
	*T. pyriformis* toxicity	0.409
	Minnow toxicity	−1.338

**Table 3:** Forward and reverse primers of the studied genes.

Gene	Primers Sequence (/5–––/3)	Accession Number
mmu-NLRP3	F:5′–TCACAACTCGCCCAAGGAGGAA–3′ R:5′–AAGAGACCACGGCAGAAGCTAG–3′	NM_145827
mmu-NF-kB P65	F:5′–CAGGACCAGGAACAGTTCGAA–3′ R:5′–CCAGGTTCTGGAAGCTATGGAT–3′	NM_009045.5
18S rRNA	F:5′–GTAACCCGTTGAACCCCATT–3′ R:5′–CAAGCTTATGACCCGCACTT–3′	NR_046237.2

## Data Availability

The original contributions presented in this study are included in the article/Supplementary Materials. Further inquiries can be directed to the corresponding authors.

## Supplementary Materials

The following supporting information can be downloaded at https://www.mdpi.com/article/10.3390/ph19050673/s1, Figure S1: FTIR analysis of 7; Figure S2: ^1^H-NMR of 7 in DMSO-d6; Figure S3: ^13^C-NMR of 7 in DMSO-d6; Figure S4: Mass spectrometry analysis of 7; Figure S5: HPLC analysis; Figure S6: Real-time PCR analysis of gene expression in brain tissue samples; Figure S7: Low magnification Histopathological examination (×200) of hippocampus section of (A) the control group I showing normal histological architecture of the hippocampus; Figure S8: High magnification Histopathological examination (×400) of hippocampus section of the hippocampal cornu Ammonis 1 region of the groups under study.

## Abbreviations

The following abbreviations are used in this manuscript:

AD	Alzheimer’s disease
CNS	Central nervous system
DEX	Dexamethasone
LPS	Lipopolysaccharide
MDA	Malondialdehyde
NO	Nitric oxide
NLRP3	NOD-like receptor family pyrin domain-containing 3
NORT	Novel Object Recognition
NF-κB	Nuclear factor kappa B
OFT	Open Field Test
SOD	Superoxide dismutase
