# Supplementary material for: Evaluation of the Potential Anti-Inflammatory Effect of a New Coumarin–Quinoline Hybrid in LPS-Induced Neuroinflammation
Source: Pharmaceuticals (Basel). 2026 Apr 25;19(5):673. doi: 10.3390/ph19050673 (PMC13210173; doi:10.3390/ph19050673)
Supplement: Supplementary file 1 [file pharmaceuticals-19-00673-s001.zip › pharmaceuticals-4169130-supplementary.pdf]

Supplementary Materials:

"New Coumarin-Quinoline Hybrid Attenuates LPS-Induced Neuroinflammation via Modulation of Inflammatory and Oxidative Pathways"

Omnia Hamdy Mohamed Shehata [1], Eman Abdelaziz [1], Hadeer Ali [1], Elshaymaa I. Elmongy [2], Reem Binsuwaidan [3], Wafaa M. Ibrahim [4]*, Sabreen El-Gamasy [1], and Ibrahim El Tantawy El Sayed [1]*

Table of Contents:

1. **Fig. S1: FTIR Analysis of 7.**
2. **Fig.S2: 1H-NMR of 7 using DMSO-d6.**
3. **Fig.S3. ^{13}C-NMR of 7 using DMSO-d6.**
4. **Fig. S4: Mass spectrometry analysis 7.**
5. **Fig.S5 (HPLC analysis)**
6. **Fig. S6. Real-time PCR analysis of gene expression**
7. **Fig. S7 Low magnification Histopathological examination (x200) of hippocampus section among the studied groups.**
8. **Fig. S8 High magnification Histopathological examination (x400) of hippocampus section of the hippocampal cornu Ammonis 1 region of the groups under study**

General

^{1}H-NMR and ^{13}C-NMR spectra were recorded on a Bruker spectrometer (Germany) in DMSO-d6 (500 MHz for 1H-NMR and 125 MHz for ^{13}C-NMR). FTIR analyses were performed with Alpha, Bruker Germany, Mass spectrometry was analyzed using Agilent G1946D LC/MS, USA, and HPLC was analyzed using Agilent 1100 liquid chromatography.

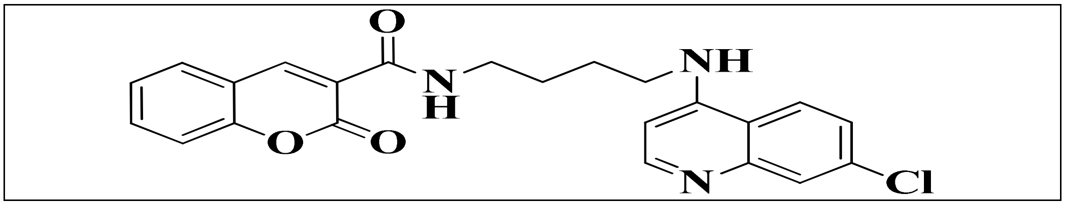

Structure of the Coumarin-quinoline hybrid 7

Figure S1: FTIR Analysis of 7

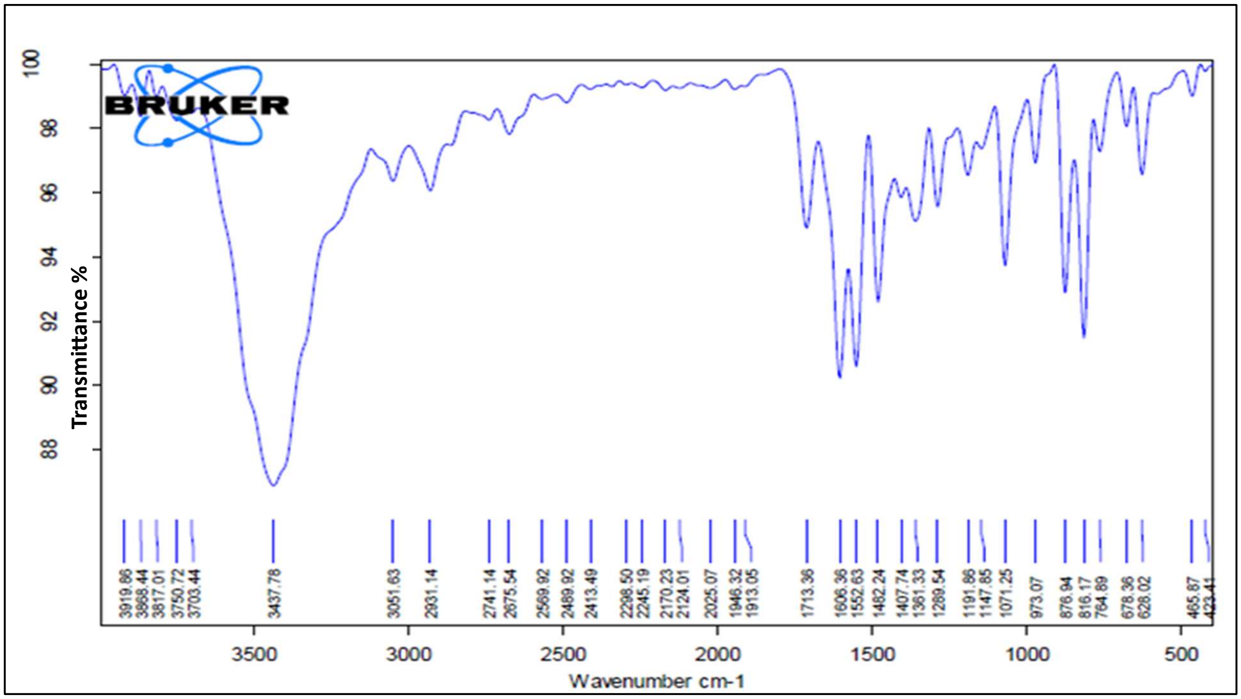

Fig.S2: ^{1}H-NMR of 7 in DMSO-d6

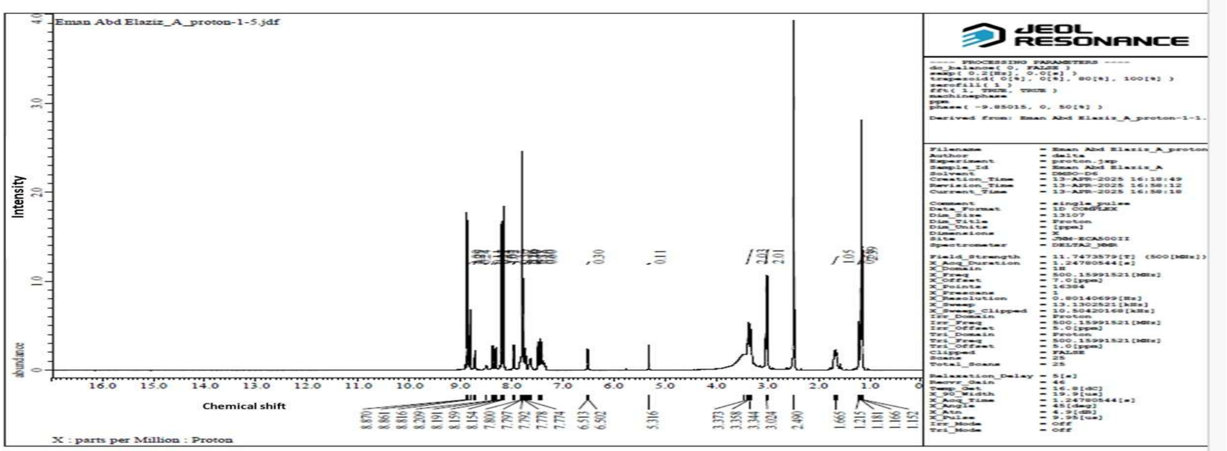

Fig.S3. ^{13}C-NMR of 7 in DMSO-d6

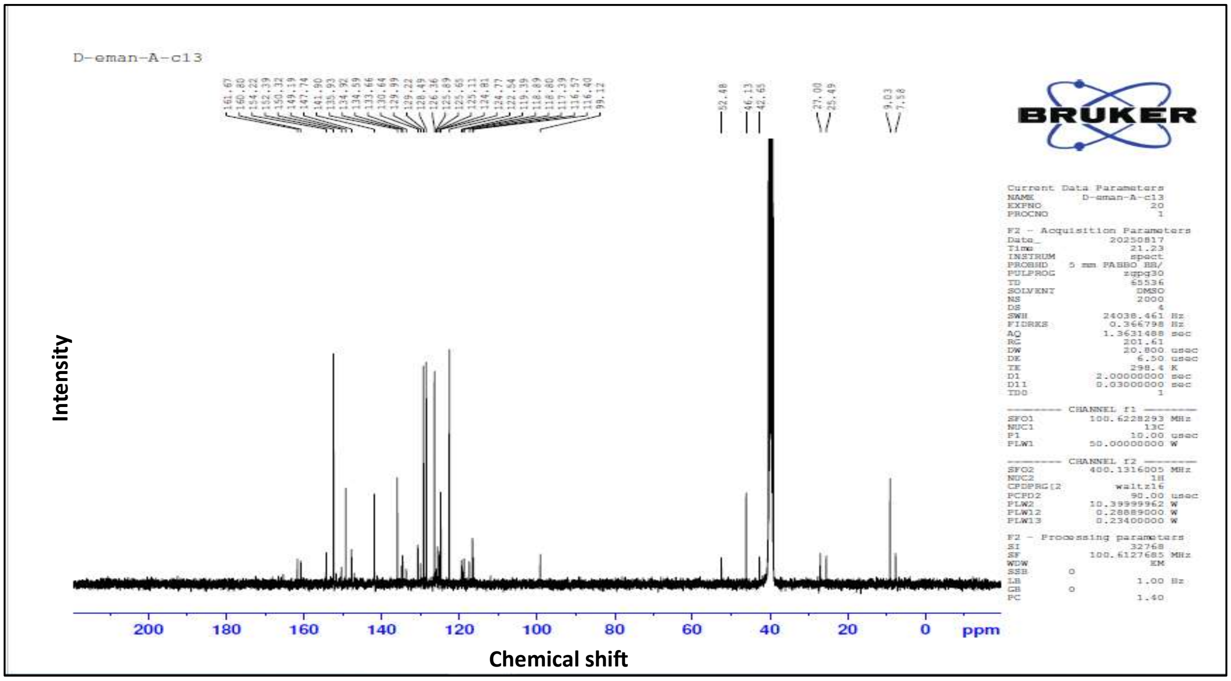

Fig. S4: Mass spectrometry analysis 7.

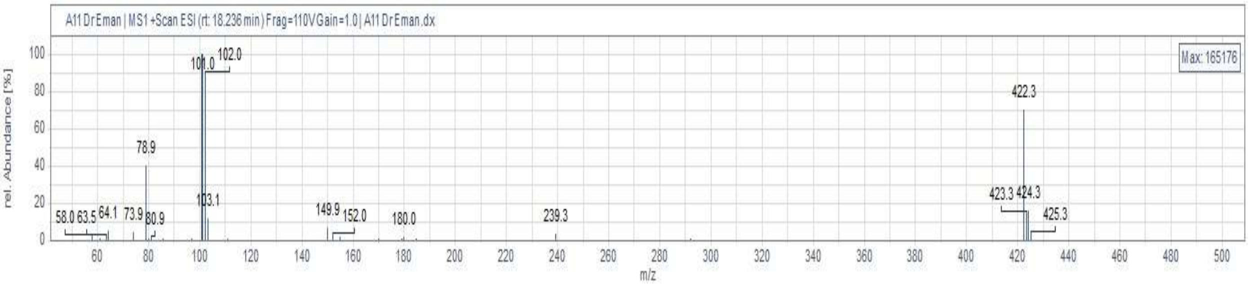

Fig.S5 (HPLC analysis)

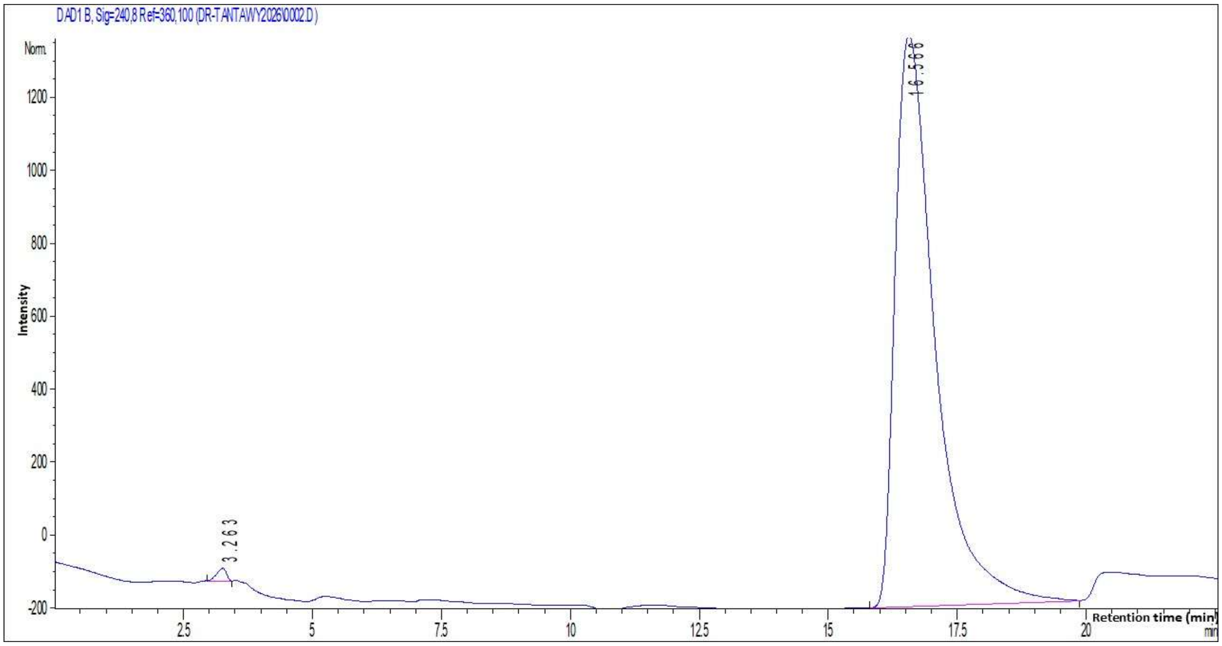

#	Time	Area	Height	Width	Symmetry	Area%
1	3.263	394.6	31	0.1947	1.676	0.515
2	16.566	76258.6	1412.3	0.7919	0.483	99.485

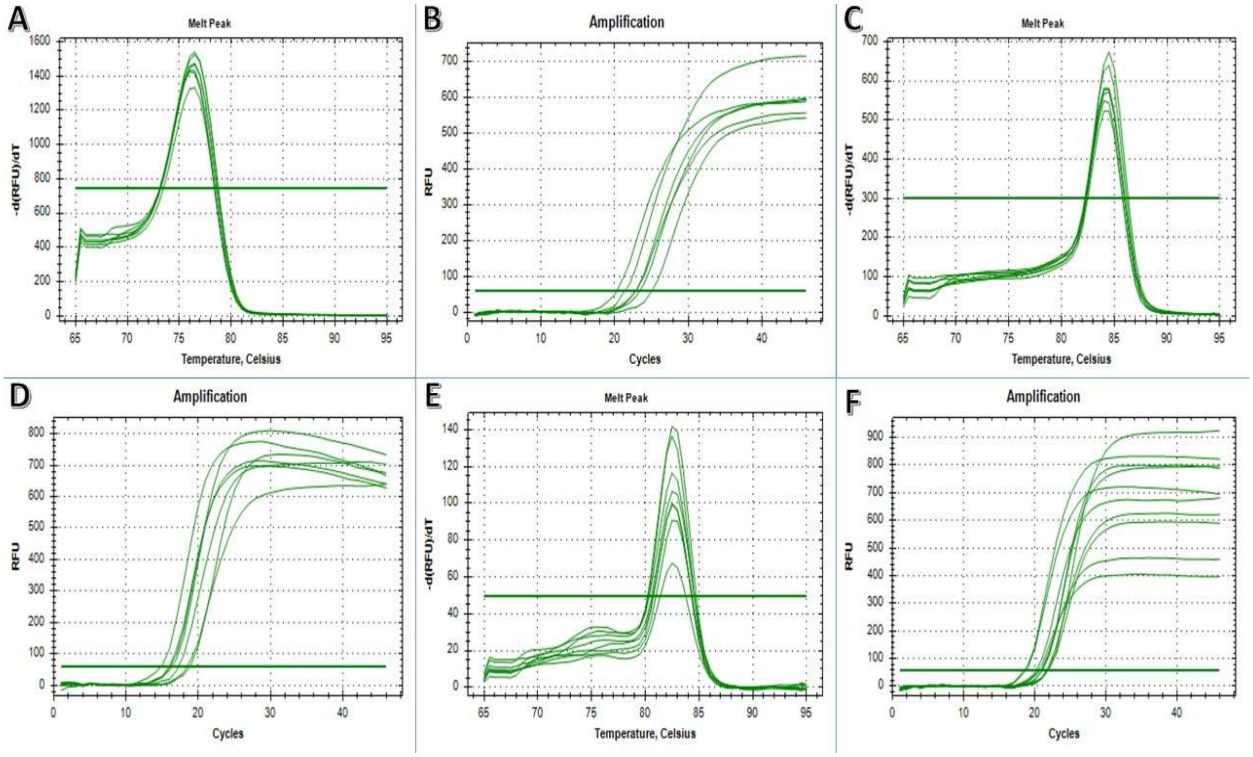

Fig. S6. Real-time PCR analysis of gene expression in brain tissue samples**. A)** Melting curve analysis of NLRP3 gene expression. **B)** Amplification curve of NLRP3 gene expression. **C)** Melting curve analysis of nuclear factor-kB (NF-kB) relative gene expression. **D)** Amplification curve of NF-kB relative gene expression. **E):** Melting curve analysis of 18s RNA used as an internal control. **F)** Amplification curves of 18s RNA used as an internal control.

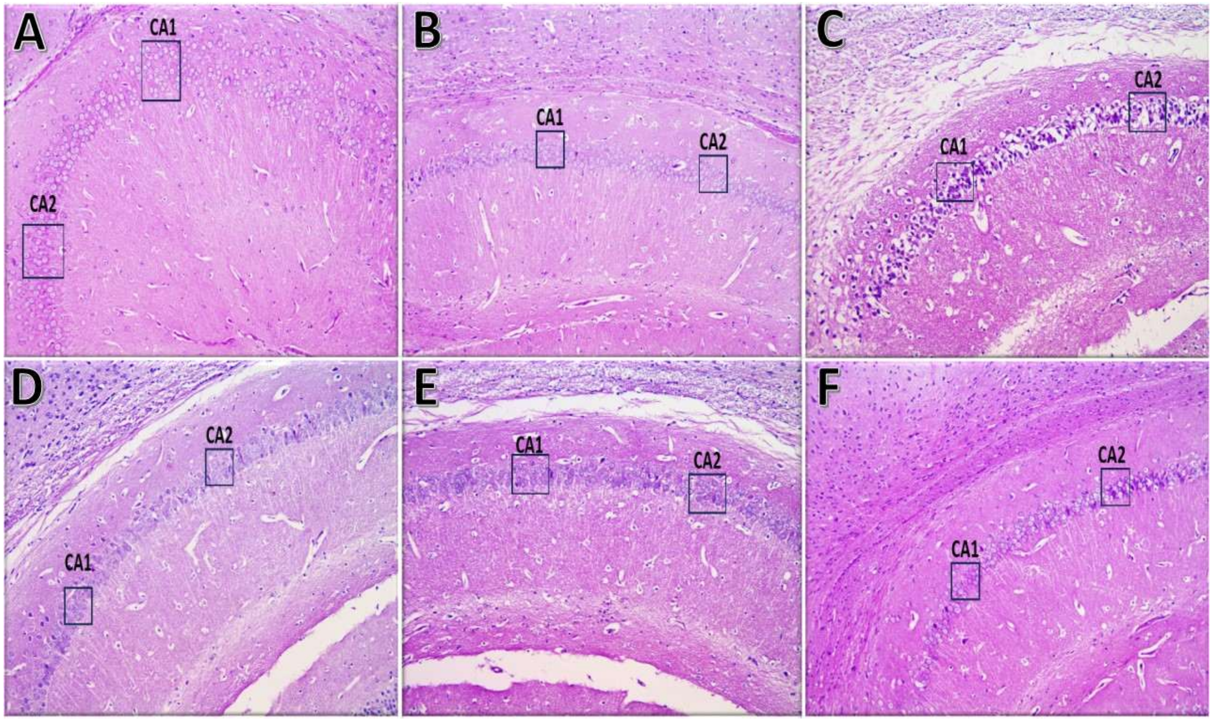

Fig. S7 Low magnification Histopathological examination (x200) of hippocampus section of A) the control group I showing normal histological architecture of the hippocampus. Cornu Ammonis (CA1 and CA2) regions are seen. B) coumarin-Quinoline hybrid group II showing normal histological architecture of the hippocampus. Cornu Ammonis (CA1 and CA2) regions are seen. C) LPS group IV showing its different regions: cornu Ammonis CA1 and CA2. D) LPS+coumarin-Quinoline hybrid group V showing its different regions: cornu Ammonis CA1 and CA2. E) LPS+ Dexa group III showing its different regions: cornu Ammonis CA1 and CA2. F) LPS+coumarin-Quinoline hybrid +Dexa group VI showing marked improvement of histological architecture; its different regions; cornu Ammonis CA1 and CA2 are seen.

(H&E Mic. Mag. x 200)

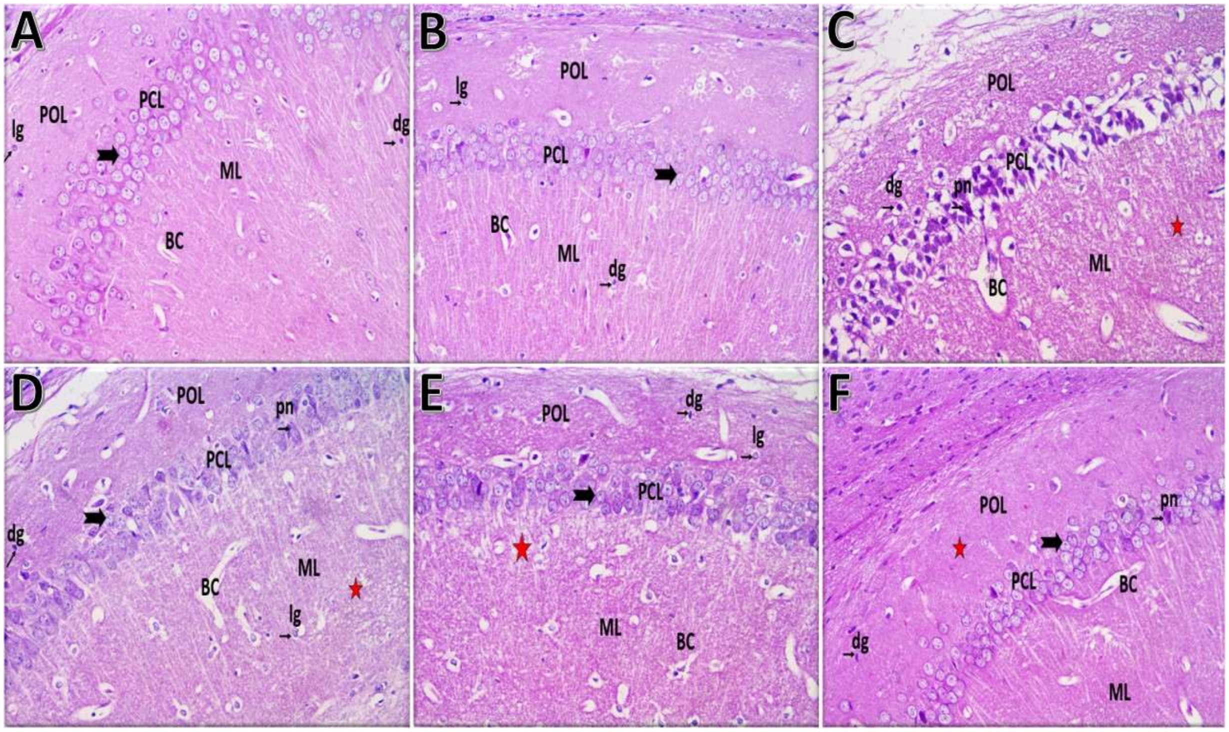

Fig. S8 High magnification Histopathological examination (x400) of hippocampus section of the hippocampal cornu Ammonis 1 region of the groups under study, showing: A) The control group revealed three distinct layers: the polymorphic layer (POL), pyramidal cell layer (PCL), and molecular layer (ML). The cell bodies of the pyramidal neurons (bifid black arrow) in PCL are closely packed, regularly arranged in three to four rows, and appear small with vesicular nuclei, prominent nucleoli, and little cytoplasm. The nuclei of glial cells with normal blood capillaries (BC) are stained deeply (dg) and lightly (lg) in the POL and ML. B) The histological structure of the new Coumarin-Quinoline Hybrid group II was normal and comparable to that of the control group. C) LPS group III displayed dilated blood capillaries (BC), perinuclear halo surrounding deeply stained glial cell nuclei (dg), vacuolated neuropils (red star), fewer pyramidal cells (PCL), and the majority of pyramidal neurons with pyknotic nuclei (pn). D) Group IV treated with LPS + new Coumarin-Quinoline Hybrid showed improvement of histological architecture like the control group, except for dilated blood capillary (BC), a few pyknotic nuclei of pyramidal cells (pn), and mild vacuolated neuropil (red star). E) LPS + Dexa group V revealed histological structure like the control group except for mild vacuolated neuropil, which is seen (red star). F) LPS + Dexa + new Coumarin-Quinoline Hybrid group VI revealed marked improvement of histological architecture, like the control group.

(H&E Mic. Mag. x 400).
